# Functional Analysis of Monkeypox and Interrelationship between Monkeypox and COVID-19 by Bioinformatic Analysis

**DOI:** 10.1155/2023/8511036

**Published:** 2023-03-23

**Authors:** Eun Jung Sohn

**Affiliations:** College of Medicine, Pusan National University, Yangsan 50612, Republic of Korea

## Abstract

The outbreak of monkeypox may be considered a novel and urgent threat after the coronavirus disease (COVID-19). No wide-ranging studies have been conducted on this disease since it was first reported. We systematically assessed the functional role of gene expression in cells infected with the monkeypox virus using transcriptome profiling and compared the functional relation with that of COVID-19. Based on the Gene Expression Omnibus database, we obtained 212 differentially expressed genes (DEGs) of GSE36854 and GSE21001 of monkeypox datasets. Enrichment analyses, including KEGG and gene ontology (GO) analyses, were performed to identify the common function of 212 DEGs of GSE36854 and GSE21001. CytoHubba and Molecular Complex Detection were performed to determine the core genes after a protein–protein interaction (PPI). Metascape/COVID-19 was used to compare DEGs of monkeypox and COVID-19. GO analysis of 212 DEGs of GSE36854 and GSE21001 for monkeypox infection showed cellular response to cytokine stimulus, cell activation, and cell differentiation regulation. KEGG analysis of 212 DEGs of GSE36854 and GSE21001 for monkeypox infection showed involvement of monkeypox in COVID-19, cytokine-cytokine receptor interaction, inflammatory bowel disease, atherosclerosis, TNF signaling, and T cell receptor signaling. By comparing our data with published transcriptome of severe acute respiratory syndrome coronavirus 2 infections in other cell lines, the common function of monkeypox and COVID-19 includes cytokine signaling in the immune system, TNF signaling, and MAPK cascade regulation. Thus, our data suggest that the molecular connections identified between COVID-19 and monkeypox elucidate the causes of monkeypox.

## 1. Introduction

Monkeypox is a zoonotic disease caused by the monkeypox virus (MPXV), belonging to the Orthopoxvirus genus, which includes double-stranded DNA viruses [1, 2]. The MPXV has been reported in Western and Central African countries, the United States, and Europe [3]. MPXV penetrates the body through direct contact via the skin, airways, or mucous membranes such as nose, eyes, or mouth from an infected person, through respiratory droplets of face-to-face contact and through indirect contact via contaminated materials [4]. The symptoms of MPXV infection are similar to those of a milder form of smallpox [5]. The distinction is that lymphadenopathy is caused by MPXV infection rather than smallpox [6]. The MPXV has split into two distinct clades: West Africa and the Congo Basin [7]. The diseases caused by the two MPXV clades differ in clinical and epidemiological characteristics. The Congo Basin strain has a 10% mortality rate [8]. West Africa has a fatality rate of about 1%, with patients with the human immunodeficiency virus (HIV) coinfection having a higher mortality rate [9].

In comparison to the West African clade, the Congo Basin monkeypox clade selectively suppresses host responses such as apoptosis and growth factor responses [10]. By repressing cognate T cell activation, MPXV avoids antiviral CD4+ and CD8+ T cell responses [11]. Realegeno et al. have shown that the high-throughput genetic screen of MPXV infection in haploid cell is involved in Golgi-associated retrograde protein complex [12]. Monkeypox requires heat shock factor 1 (HSF1) for infection [13]. MPXV infection resulted in significant increases in the number of natural killer cells and cell proliferation [14].

The outbreak of coronavirus disease (COVID-19) caused by the severe acute respiratory syndrome coronavirus 2 (SARS-CoV-2) has spread globally in December 8, 2020, according to the World Health Organization [15]. COVID-19 has affected in various diseases such as inflammatory response and nervous system symptoms such as ischemic stroke and delineation [16, 17]. Molecular and immunological diagnostic techniques have been developed to identify SARS-CoV-2 infection [18]. Currently, the most reliable method for diagnosing SARS-CoV-2 is real-time PCR-based assays for viral RNA detection [18, 19]. Furthermore, more advanced molecular approaches for detecting SARS-CoV-2 RNA are being developed, including next-generation sequencing (NGS), droplet-digital PCR (ddPCR), and clustered regularly interspaced short palindromic repeats (CRISPRs) [18, 20–22]. Recently, the number of monkeypox cases has increased in 2022 after the COVID-19 outbreak; however, the natural reservoir and function of monkeypox are unknown.

Several studies have reported the possible relationship between monkeypox and COVID-19. One connection is that patients with SARS-CoV-2 infection are more prone to coinfect with MPXV due to the reduced amount of circulating immune cells [23]. Another link between the SARS-CoV-2 and MPXV outbreaks is a lack of immunity in younger generations to the smallpox virus. Smallpox is in the same family as monkeypox, and the discontinuation of its vaccine contributes to increased monkeypox cases as well as spread of SARS-CoV-2 by lowering immunity among local residents [23, 24]. It is still unclear whether the new monkeypox outbreak is a distinct phenomenon or if it is exacerbated by the COVID-19 pandemic [25]. Because of the current small patient sample size, the exact cause-effect relationship between SARS-CoV-2 and MPXV coinfection cannot yet be established [25, 26].

In this study, we aimed to investigate the possible molecular function of monkeypox by performing bioinformatic analyses. In addition, by comparing our data of monkeypox infection with published transcriptome of SARS-CoV-2 infection in other cell lines, we found that the function of monkey pox and COVID-19 infection is similar in terms of cytokine signaling in the immune system and TNF signaling. Therefore, the potential functional link of monkeypox and COVID-19 is described in this bioinformatic study.

## 2. Materials and Methods

### 2.1. The Collection of Databases and the Identification of DEGs

To create a dataset of monkeypox infection, DEGs of monkeypox infection were obtained from the Gene Expression Omnibus (GEO, https://www.ncbi.nlm.nih.gov/geo/) database. Two GEO datasets were collected to obtain the DEGs related to monkeypox, including GSE21001 and GSE36854, and the datasets were analyzed using the GEO2R (https://www.ncbi.nlm.nih.gov/geo/geo2r/) web tool. GSE21001 was the database for GeneChip rhesus macaque genome microarrays on a 3- and 7-hour postinfection (hpi) time point after an MPXV infection on *Macaca mulatta* kidney epithelial cells (MK2) [27]. GSE36854 was the database for the gene expression profile at 6 hpi after infection with MPXV strain MSF#6 on HeLa cells [28]. The cutoff criteria were obtained for GSE21001 and GSE36854 using an adjusted *P* value <0.05.

### 2.2. Gene Ontology (GO) and KEGG Pathway Enrichment Analyses of DEGs of Monkeypox

To examine the enrichment analysis of 212 common differentially expressed genes (DEGs) from the GSE21001 and GSE36854 datasets of MPXV infection, KEGG pathway and GO analyses were performed using the web tool Shiny Go (ShinyGO 0.76 (https://sdstate.edu)) [29, 30]. For the enrichment biological pathways of the comparison between monkeypox and COVID-19, Metascape/COVID analysis (https://metascape.org/COVID) was carried out [31].

### 2.3. Identify Hub Genes of MPXV Infection

The cytoHubba plugins of Cytoscape were used to identify the top 20 nodes (hub genes), ranked using the maximal clique centrality (MCC) algorithm of cytoHubba. The Metascape tool [31] was to identify the Molecular Complex Detection (MCODE) components for functional gene clusters.

### 2.4. DisGeNET, PaGenBase, and TRRUST for Enrichment Analyses

DisGeNET, PaGenBase, and TRRUST were used to analyze the monkeypox infection, and comparison between monkeypox and COVID-19 was performed through Metascape (https://metascape.org). DisGeNET is a platform for the genetic underpinning of human diseases [32, 33]. PaGenBase is a database for obtaining information on pattern genes under various tissue and time specific conditions [34]. TRRUST is a platform for transcriptional regulatory networks [35].

## 3. Results

### 3.1. Design

**Figure 1:**
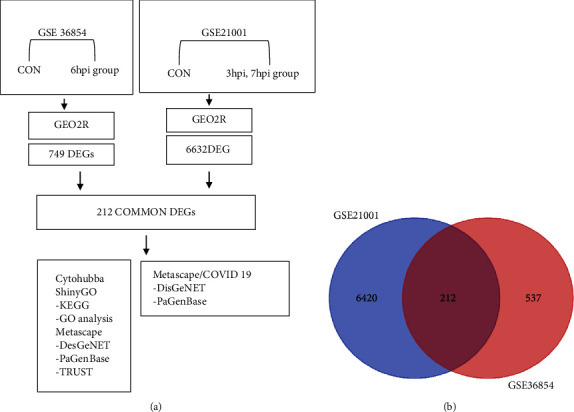
Flowchart of the present study. (a) Study design of the present study. DEG, differentially expressed genes; GO, gene ontology. (b) A Venn diagram between 212 DEGs of GSE21001 and GSE36854. 212 DEGs are shared across the GSE21001 and GSE36854 datasets. hpi, hour postinfection.

To determine the function of the MPXV infection, the public datasets GSE36854 and GSE21001 were used.  Figure 1(a) shows a flowchart of this study. GEO2R was used to obtain DEGs from the GEO database. Adjusted *P* values <0.05 were considered as the criteria for the DEGs.  Figure 1(b) shows the intersections of the two groups of GSE36854 and GSE21001, indicating the common DEGs.

### 3.2. Screening of Hub Genes and Important Modules of MPXV Infection

**Figure 2:**
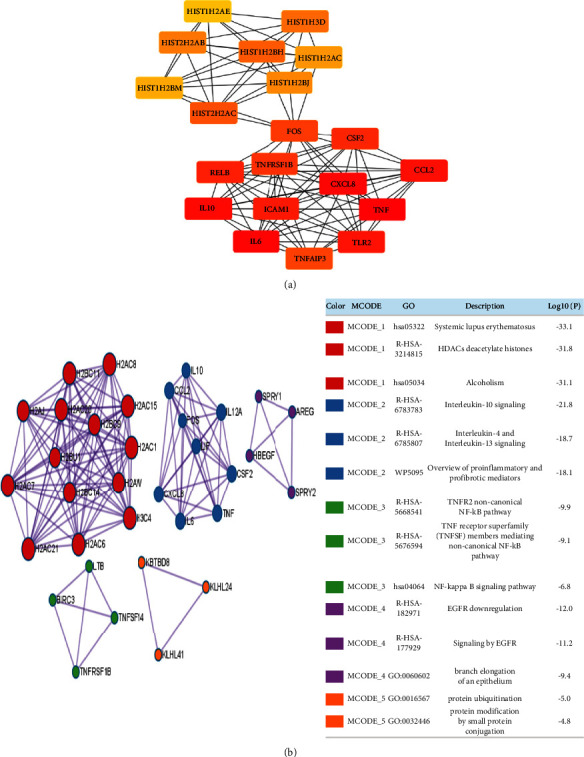
CytoHubba gene and MCODE enrichment analyses of monkeypox infection. (a) Analysis of the hub genes from 212 DEGs of monkeypox infection datasets (GSE21001 and GSE36854) from the PPI network with maximal clique centrality (MCC) algorithm in cytoHubba. Edges represent the protein-protein associations and display the protein-protein links. The red nodes show genes with a high MCC score. (b) The MCODE networks from 212 DEGs of monkeypox datasets of GSE21001 and GSE36854. The MCODE networks were analyzed using Metascape (https://metascape.org), which was accessed on May 11, 2022.

We constructed the protein-protein interaction networks for 211 DEGs of GSE36854 and GSE21001 of the MPXV infection using the Cytoscape and cytoHubba plugins. Twenty hub genes from 211 DEGs of GSE36854 and GSE21001, including HIST1H2AE, HIST2H2A B, HST1H2BM, HIST2H2AC, HIST1H3D, HIST1H2BH, HIST1H2AC, HIST1H2BJ, FOS, CSF2, RELB, IL-10, TNFRSF1B, CXCL8, ICAM1, CCL2, TNF, IL-6, TLR2, and TNFAIP3, were obtained (Figure 2(a)).

To obtain the functional clusters of MPXV infection, a module analysis was carried out using the Metascape tool. In the MCODE analysis by Metascape, 211 DEGs of GSE36854 and GSE21001 of MPXV infection were grouped into five modules. The pathways in module 1 were mainly associated with systemic lupus erythematosus, histone deacetylases (HDACs), and alcoholism. Interleukin -10, -4, -13 signaling, and overview of proinflammatory and profibrotic mediators were involved in module 2. The MCODE3 module included TNFR2 noncanonical NF-kB pathway, TNF receptor superfamily (TNFSF) members mediating noncanonical NF-kB pathway, and NF-kappa B signaling pathway. The MCODE4 module included EGFR downregulation, signaling by EGFR, and branch elongation of an epithelium. Finally, the MCODE5 module included protein ubiquitination and protein modification by small protein conjugation (Figure 2(b)).

### 3.3. KEGG and GO Analyses of MPXV Infection

**Figure 3:**
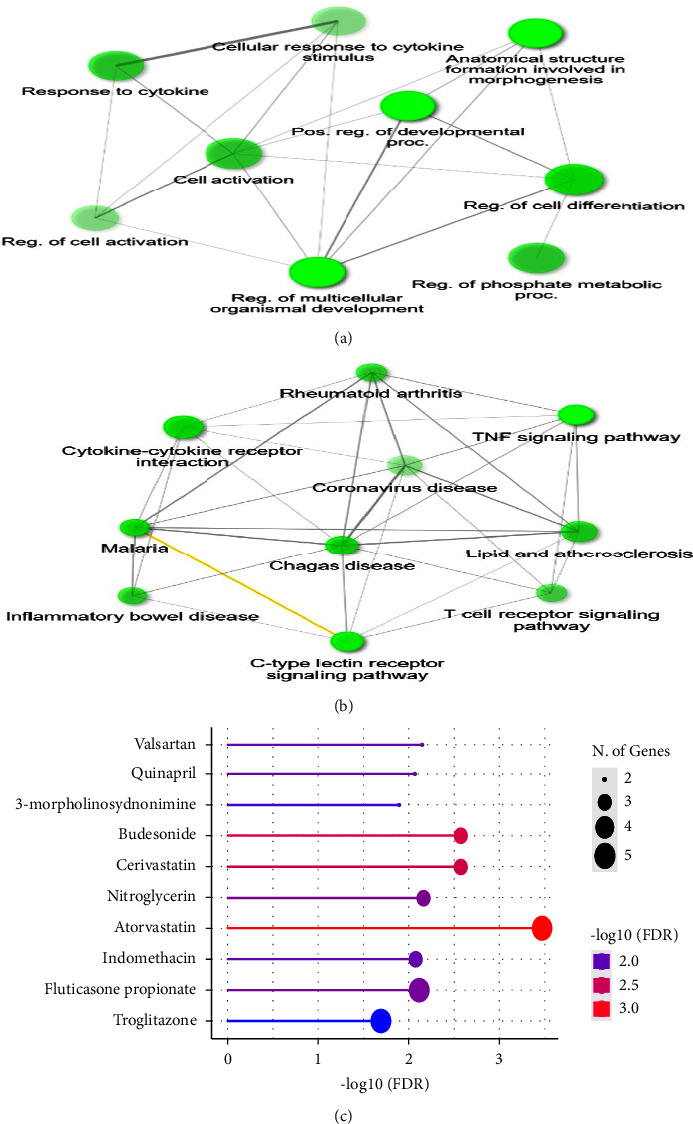
Gene ontology enrichment analysis and interaction of 212 DEGs between GSE36854 and GSE21001 of monkeypox infection. A network of GO BP terms (a) and KEGG pathway (b) of 212 DEGs of GSE36854 and GSE21001 of monkeypox infection by using the SHINEY GO 0.76 online tool (https://bioinformatics.sdstat.edu). (c) The top ten drugs related to monkeypox using 212 DEGs of GSE36854 and GSE21001 for MPXV infection by drug matador from the SHINEY GO 0.76 online tool.

To determine the functional role of MPXV infection, the KEGG and GO analyses of 212 DEGs of GSE36854 and GSE21001 were carried out with the online tool SHINEY GO (https://bioinformatics.sdstate.edu/go/). The network of GO BP of 211 DEGs from the MPXV infection using Shiny Go was mainly involved in the regulation of multicellular organism development, regulation of phosphate metabolic process, regulation of cell differentiation, and positive regulation (Figure 3(a)). As shown in Figure 3(b), KEGG analysis of 212 DEGs from GSE36854 and GSE21001 showed involvement in coronavirus disease, cytokine-cytokine receptor interaction, inflammatory bowel disease, Chagas disease, lipid and atherosclerosis, T cell receptor signaling pathway, C-type lectin receptor signaling pathway, rheumatoid arthritis, and malaria.

### 3.4. Predicted Drug-Target Relationship Using 212 DEGs of Monkeypox Infection

We used the drug matador from the SHINEY GO online tool to predict drug-target relationships using 212 DEGs of GSE36854 and GSE21001 for MPXV infection. The top ten drugs related to monkeypox include troglitazone, fluticasone propionate, indomethacin, atorvastatin, nitroglycerin, cerivastatin, budesonide, 3-morpholinosydnonimine, quinapril, and valsartan (Figure 3(c)).

### 3.5. DisGNET, PaGenBase, and TRRUST of 212 DEGs from MPXV Infection

**Figure 4:**
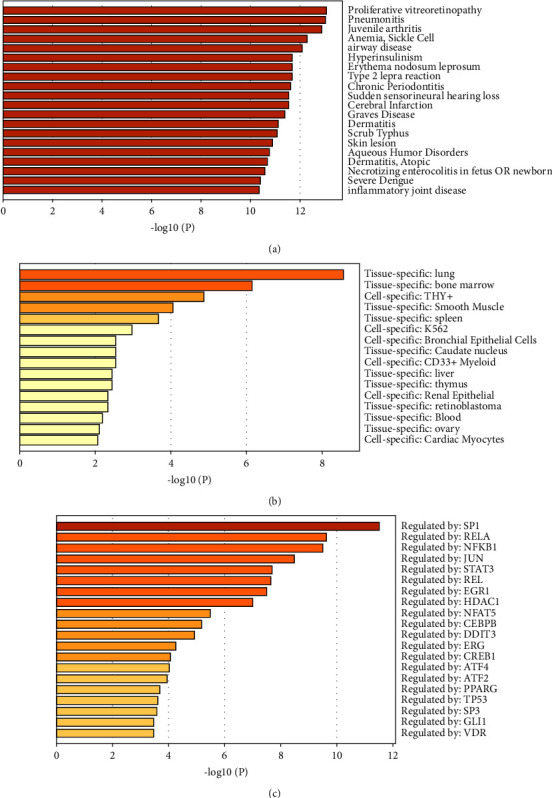
DisGeNET, TRRUST, and PaGenBase of monkeypox infection. (a) DisGeNET database of 212 DEGs of GSE36854 and GSE21001 for enrichment analyses of diseases. (b) TRRUST database of 212 DEGs of GSE35854 and GSE21001 for enrichment of transcriptional regulators. (c) PaGenBase database for tissue characteristics of 212 DEGs of GSE36854 and GSE21001.

DisGeNET database disease enrichment analysis by using the Metascape tool demonstrated that the hub genes of 212 DEGs from GSE36854 and GSE21001 were associated with proliferative vitreoretinopathy, pneumonitis, juvenile arthritis, anemia, airway disease, and so on (Figure 4(a)). PaGenBase was used to perform a tissue characteristic enrichment analysis with the Metascape tool and showed that the hub genes of 212 DEGs from GSE36854 and GSE21001 were mainly enriched in the lung (Figure 4(b)). The TRRUST database analysis by using the Metascape tool demonstrated that RELA, SP1, and NF-*κ*B1 are core transcription factors regulating the hub genes of 212 DEGs of MPXV infection (Figure 4(c)).

### 3.6. The Functional Analysis of MPXV and SARS-CoV-2 Infections

**Figure 5:**
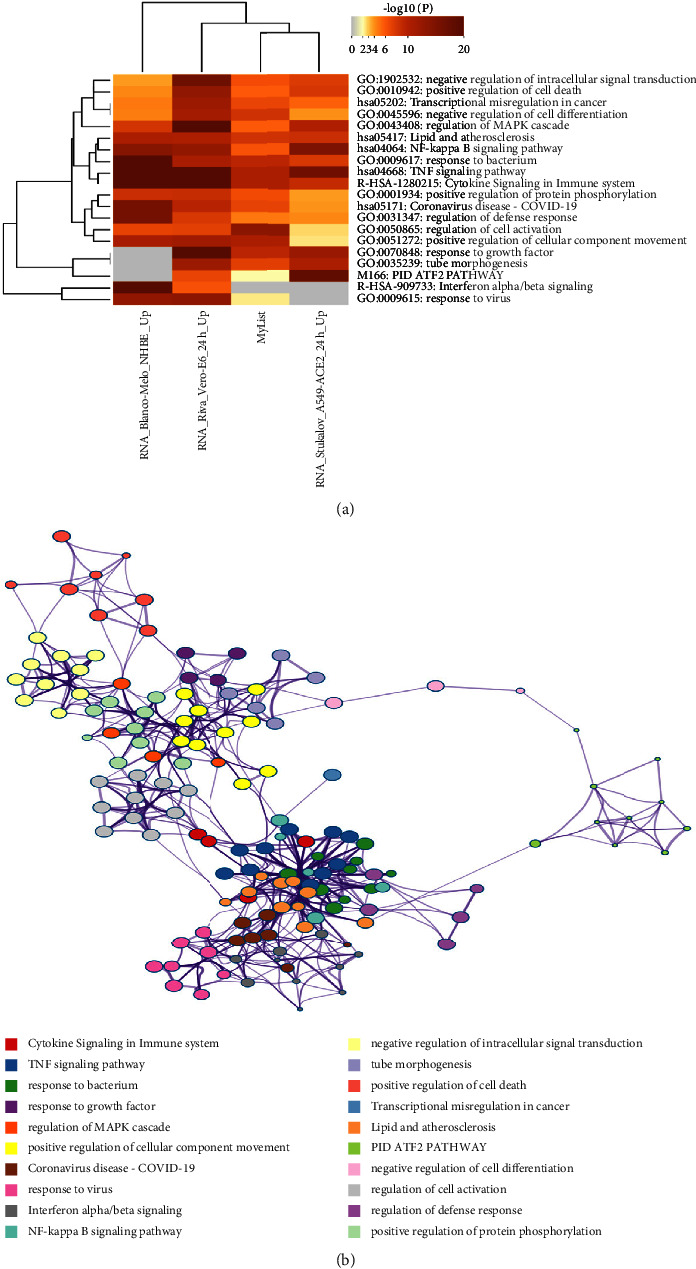
Comparison of biological function between monkeypox and COVID-19. (a) Hierarchical clustering of the enriched terms of 212 DEGs of GSE36854 and GSE21001 from monkeypox (my list) and as compared to COVID-19 cell lines analyzed through the metascape database. The metascape/COVID-19 database was accessed on May 11, 2022. (b) Network plot of enriched terms for DEGs of monkeypox infection compared to SARS-CoV-2 infection cell lines analyzed through the metascape database.

KEGG analysis of 212 DEGs from GSE36854 and GSE21001 for MPXV infection revealed COVID-19 relationship with COVID-19. Therefore, to determine the relationship between MPXV and SARS-CoV-2 infections, the Coronascape COVID database (https://metascape.org/COVID) was utilized. The biological function of coexpressed genes between SARS-CoV-2 infection and 212 DEGs from GSE36854 and GSE21001 for MPXV infection included GO1902532 negative regulation of intracellular signal transduction, GO0010942 positive regulation of cell death, hsa05202 transcriptional misregulation in cancer, GO0045596 negative regulation of cell differentiation, GO0043408 regulation of the MPAK cascade, hsa05417 NF-kappa B signaling pathway, GO 0009617 response to bacterium, and hsa04668 TNF signaling pathway (Figure 5(a)).

The network was visualized using Cytoscape5, wherein each node represents an enriched term and was colored first by its cluster ID and MCODE algorithm to see densely connected network components of genes coexpressed between SARS-CoV-2 and MPXV infections, as shown in Figure 5(b). The PPI interactions of the coexpressed genes were subjected to pathway and process enrichment analyses. The network of enriched term by clusters is involved in cytokine signaling in the immune system, TNF signaling, response to growth factor, regulation of MAPK cascade, positive regulation of cellular components, COVID-19, and so on (Figure 5(b)).

### 3.7. Key Transcription Factors and Associated Human Disease by Comparing between MPXV and SARS-CoV-2 Infections in the Cells

**Figure 6:**
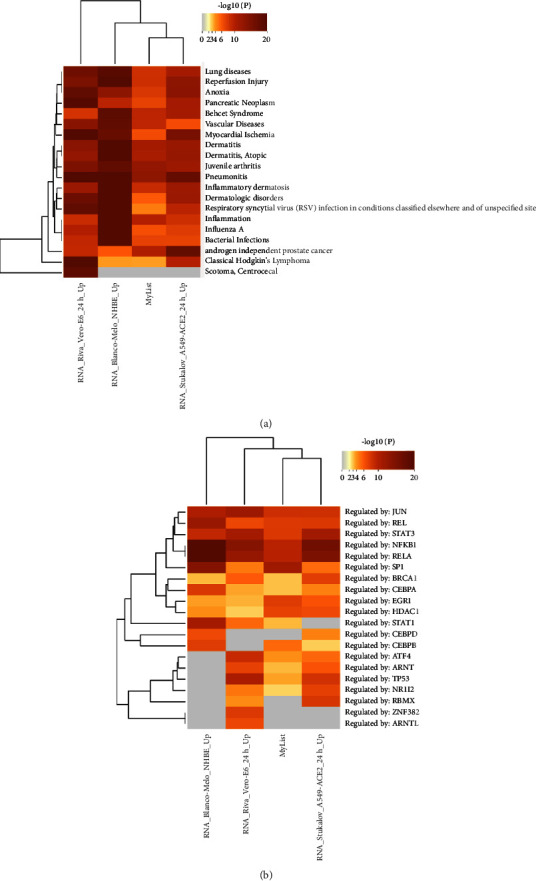
Comparison of the results of the DisGeNET and TRRUST analyses between monkeypox and SARS-CoV-2 infections. (a) DisGeNET database was used in the comparison between 212 DEGs of GSE36854 and GSE21001 from monkeypox infection (my list) and COVID-19 by Metascape/COVID-19 for enrichment analysis of diseases. (b) TRRUST database used for enrichment of transcriptional regulators; results of monkeypox and COVID-19 were compared using Metascape/COVID-19, which was accessed on May 11, 2022. My list means 212 DEGs from GSE36854 and GSE21001 for MPXV infection.

The DisGeNET discovery platform (https://www.disgenet.org) provided the Coronascape, a COVID-19 database (https://metascape.org/COVID), to determine human diseases associated with COVID-19 and monkeypox. We used 212 DEGs from GSE36854 and GSE21001 to compare the MPXV and SARS-CoV-2 infections. DisGeNET database disease enrichment analysis of our data (212 DEGs from GSE36854 and GSE21001 for MPXV infection) and COVID-19 database demonstrated that the hub genes were associated with lung disease, reperfusion injury, anoxia, dermatitis, pneumonitis, inflammatory dermatosis, and others (Figure 6(a)). Analysis of the transcription factors using the TRRUST database from our data (212 DEGs from GSE36854 and GSE21001) for MPXV infection and COVID-19 database by Metascape identified JUN, REL, STAT3, NFKB1, RELA, SP1, and so on (Figure 6(b)).

## 4. Discussion

The outbreak of monkeypox has spread across several countries in 2022 after the COVID-19 pandemic. However, there is no information of the function of monkeypox and its relation with COVID-19. In the present study, the function of MPXV infection was identified by bioinformatics analyses. In addition, key pathways between SARS-CoV-2 and monkeypox infections were suggested using the bioinformatics analyses tool.

Although SARS-CoV-2 and MPXV are both enveloped viruses that replicate in the cytoplasm and require host machinery to replicate and make new viral proteins, there are distinctions between the two viruses [23]. MPXV is a double-stranded DNA virus, whereas SARS-CoV-2 is a single-stranded RNA virus. Within the last two years, SARS-CoV-2 has several different clades being mutated from its alpha form to beta, gamma, delta, and omicron clades [23, 36]. MPXV has two clades such the Congo Basin and Nigerian clades [6]. MPXV is transmitted primarily through very close physical contact from human-human transmission and long-term contact with an infected animal, whereas COVID-19 illness is caused by droplet, direct contact, and airborne transmission of SARS-CoV-2 [23]. The SARS-CoV-2 pandemic spreads in the form of tides and waves, whereas MPXV spreads more linearly [23]. MPXV enters cells via macropinocytosis, whereas SARS-CoV-2 penetrates the human body by attaching its spikes to endothelial cells angiotensin converting enzyme 2 receptors (ACE-2) [23].

In this study, the hub genes of 212 DEGs of MPXV infection by cytoHubba analysis were involved in cytokine-related genes such IL-6, IL-10, TNF, CCL2, ICAM1, CXCL8, and HDAC-related genes. In addition, the MCODE analysis also associated with systemic lupus erythematosus, HDACs, and IL‐10 and ‐4 signaling. HDACs and histone acetyl transferases play major roles in cell survival, growth, and proliferation as well as the regulation of gene transcription [37]. IL-10 plays an important role as an anti-inflammatory cytokine by preventing pathogenic infection and excessive immune response [38, 39], and IL-4 regulates the immune responses [40]. Systemic lupus erythematosus is well known as a chronic autoimmune disease that produces autoantibodies and affects multiple organs [41].

In the present study, KEGG analysis of 212 DEGs of GSE36854 and GSE21001 for MPXV infection showed its involvement in COVID-19, cytokine-cytokine receptor interaction, and TNF signaling. In addition, the biological function of coexpressed genes between SARS-CoV-2 and MPXV infections in the cell lines by Metascape/COVID-19 analysis was enriched in cytokine signaling in the immune system, TNF signaling, response to bacterium, response to growth factor, and regulation of the MAPK cascade. There is evidence that cytokine modulation is associated with the severity of the monkeypox disease in humans [42]. MPXV infection in Hela cells involves genes for proinflammatory cytokine and leukocyte chemotaxis [28] and that in MK2 cells enhanced the IL-8 gene [27, 43]. Many studies have suggested that SARS-CoV-2 is associated with a dysregulated inflammatory response, such as cytokine storm and inflammatory molecules [44, 45]. Therefore, monkeypox and COVID-19 might be associated with the inflammatory immune response.

A previous study demonstrated that transcription factors play an important role in the regulation of transcription, metabolism, and immune response [43, 46], and they are closely associated with diseases [47]. In this study, JUN, REL, NFKB1, and STAT3 are mainly involved transcriptional factor according to the TRRUST analysis when comparing MPXV and SARS-CoV-2 infections. There is evidence that JUN, REL, NFKB1, and STAT3 are involved in inflammation [48–51].

## 5. Conclusions

In conclusion, the hub genes based on the cytoHubba and MCODE analyses of MPXV infection included cytokine and HDAC-related genes. KEGG analysis of MPXV infection showed involvement of MPXV in COVID-19, cytokine-cytokine receptor interaction, and TNF signaling pathway. Our study data suggest that the function of monkeypox is similar to that COVID-19 in terms of cytokine signaling in the immune system and TNF signaling. Our study provides pathological insight of monkeypox and molecular connections between COVID-19 and monkeypox. Our study has some limitations due to the lack of experimental verification of computational data. It will inevitably need in-depth research in the future.

## Data Availability

Data used in this study are available at the NCBI (National Center for Biotechnology Information Gene Expression Omnibus), https://www.ncbi.nlm.nih.gov/geo/.

## References

[1] Isidro J., Borges V., Pinto M. (2022). Addendum: p. *Nature Medicine*.

[2] Faye O., Pratt C. B., Faye M. (2018). Genomic characterisation of human monkeypox virus in Nigeria. *The Lancet Infectious Diseases*.

[3] Adler H., Gould S., Hine P. (2022). Clinical features and management of human monkeypox: a retrospective observational study in the UK. *The Lancet Infectious Diseases*.

[4] Murphy S. (2022). Monkeypox. *British Dental Journal*.

[5] Jayswal S., Kakadiya J. (2022). A narrative review of pox: smallpox vs. monkeypox. *Egypt J Intern Med*.

[6] Kumar N., Acharya A., Gendelman H. E., Byrareddy S. N. (2022). The 2022 outbreak and the pathobiology of the monkeypox virus. *Journal of Autoimmunity*.

[7] Likos A. M., Sammons S. A., Olson V. A. (2005). A tale of two clades: monkeypox viruses. *Journal of General Virology*.

[8] Doshi R. H., Guagliardo S. A. J., Doty J. B. (2017). Epidemiologic and ecologic investigations of monkeypox, likouala department, republic of the Congo. *Emerging Infectious Diseases*.

[9] Ray S., Maunsell J. H. R. (2011). Different origins of gamma rhythm and high-gamma activity in macaque visual cortex. *PLoS Biology*.

[10] Kindrachuk J., Arsenault R., Kusalik A. (2012). Systems kinomics demonstrates Congo Basin monkeypox virus infection selectively modulates host cell signaling responses as compared to West African monkeypox virus. *Molecular and Cellular Proteomics*.

[11] Hammarlund E., Dasgupta A., Pinilla C., Norori P., Fruh K., Slifka M. K. (2008). Monkeypox virus evades antiviral CD4+ and CD8+ T cell responses by suppressing cognate T cell activation. *Proceedings of the National Academy of Sciences of the U S A*.

[12] Realegeno S., Puschnik A. S., Kumar A. (2017). Monkeypox virus host factor screen using haploid cells identifies essential role of GARP complex in extracellular virus formation. *Journal of Virology*.

[13] Filone C. M., Caballero I. S., Dower K. (2014). The master regulator of the cellular stress response (HSF1) is critical for orthopoxvirus infection. *PLoS Pathogens*.

[14] Song H., Josleyn N., Janosko K. (2013). Monkeypox virus infection of rhesus macaques induces massive expansion of natural killer cells but suppresses natural killer cell functions. *PLoS One*.

[15] Bhimraj A., Morgan R. L., Shumaker A. H. (2022). Lessons learned from coronavirus disease 2019 (COVID-19) therapies: critical perspectives from the infectious diseases society of America (IDSA) COVID-19 treatment guideline panel. *Clinical Infectious Diseases*.

[16] Nordvig A. S., Fong K. T., Willey J. Z. (2021). Potential neurologic manifestations of COVID-19. *Neurol Clin Pract*.

[17] Ramos-Casals M., Brito-Zeron P., Mariette X. (2021). Systemic and organ-specific immune-related manifestations of COVID-19. *Nature Reviews Rheumatology*.

[18] Rotondo J. C., Martini F., Maritati M. (2022). Advanced molecular and immunological diagnostic methods to detect SARS-CoV-2 infection. *Microorganisms*.

[19] Kevadiya B. D., Machhi J., Herskovitz J. (2021). Diagnostics for SARS-CoV-2 infections. *Nature Materials*.

[20] Hou T., Zeng W., Yang M. (2020). Development and evaluation of a rapid CRISPR-based diagnostic for COVID-19. *PLoS Pathogens*.

[21] Xu J., Kirtek T., Xu Y. (2021). Digital droplet PCR for SARS-CoV-2 resolves borderline cases. *American Journal of Clinical Pathology*.

[22] Chen X., Kang Y., Luo J. (2021). Next-generation sequencing reveals the progression of COVID-19. *Frontiers in Cellular and Infection Microbiology*.

[23] Roushdy T. (2022). SARS-CoV-2 and monkeypox: what is common and what is not in a present pandemic versus a potential one-a neuropsychiatric narrative review. *The Egyptian Journal of Neurology, Psychiatry and Neurosurgery*.

[24] Haider N., Guitian J., Simons D. (2022). Increased outbreaks of monkeypox highlight gaps in actual disease burden in Sub-Saharan Africa and in animal reservoirs. *International Journal of Infectious Diseases*.

[25] Farahat R. A., Abdelaal A., Shah J. (2022). Monkeypox outbreaks during COVID-19 pandemic: are we looking at an independent phenomenon or an overlapping pandemic?. *Annals of Clinical Microbiology and Antimicrobials*.

[26] El-Qushayri A. E., Reda A., Shah J. (2022). COVID-19 and monkeypox co-infection: a rapid systematic review. *Frontiers in Immunology*.

[27] Alkhalil A., Hammamieh R., Hardick J., Ichou M. A., Jett M., Ibrahim S. (2010). Gene expression profiling of monkeypox virus-infected cells reveals novel interfaces for host-virus interactions. *Virology Journal*.

[28] Bourquain D., Dabrowski P. W., Nitsche A. (2013). Comparison of host cell gene expression in cowpox, monkeypox or vaccinia virus-infected cells reveals virus-specific regulation of immune response genes. *Virology Journal*.

[29] Ge S. X., Jung D., Yao R. (2020). ShinyGO: a graphical gene-set enrichment tool for animals and plants. *Bioinformatics*.

[30] Kanehisa M., Furumichi M., Sato Y., Ishiguro-Watanabe M., Tanabe M. (2021). KEGG: integrating viruses and cellular organisms. *Nucleic Acids Research*.

[31] Zhou Y., Zhou B., Pache L. (2019). Metascape provides a biologist-oriented resource for the analysis of systems-level datasets. *Nature Communications*.

[32] Pinero J., Queralt-Rosinach N., Bravo A. (2015). DisGeNET: a discovery platform for the dynamical exploration of human diseases and their genes. *Database*.

[33] Pinero J., Bravo A., Queralt-Rosinach N. (2017). DisGeNET: a comprehensive platform integrating information on human disease-associated genes and variants. *Nucleic Acids Research*.

[34] Pan J. B., Hu S. C., Shi D. (2013). PaGenBase: a pattern gene database for the global and dynamic understanding of gene function. *PLoS One*.

[35] Han H., Shim H., Shin D. (2015). TRRUST: a reference database of human transcriptional regulatory interactions. *Scientific Reports*.

[36] Ding K., Jiang W., Xiong C., Lei M. (2022). Turning point: a new global COVID-19 wave or a signal of the beginning of the end of the global COVID-19 pandemic?. *Immunity, Inflammation and Disease*.

[37] Wang C., Fu M., Mani S., Wadler S., Senderowicz A. M., Pestell R. G. (2001). Histone acetylation and the cell-cycle in cancer. *Frontiers in Bioscience*.

[38] Ni G., Zhang L., Yang X. (2020). Targeting interleukin-10 signalling for cancer immunotherapy, a promising and complicated task. *Human Vaccines and Immunotherapeutics*.

[39] Verma R., Balakrishnan L., Sharma K. (2016). A network map of Interleukin-10 signaling pathway. *J Cell Commun Signal*.

[40] Nelms K., Keegan A. D., Zamorano J., Ryan J. J., Paul W. E. (1999). The IL-4 receptor: signaling mechanisms and biologic functions. *Annual Review of Immunology*.

[41] Pan L., Lu M. P., Wang J. H., Xu M., Yang S. R. (2020). Immunological pathogenesis and treatment of systemic lupus erythematosus. *World J Pediatr*.

[42] Johnston S. C., Johnson J. C., Stonier S. W. (2015). Cytokine modulation correlates with severity of monkeypox disease in humans. *Journal of Clinical Virology*.

[43] Singh H., Khan A. A., Dinner A. R. (2014). Gene regulatory networks in the immune system. *Trends in Immunology*.

[44] Mulchandani R., Lyngdoh T., Kakkar A. K. (2021). Deciphering the COVID-19 cytokine storm: systematic review and meta-analysis. *European Journal of Clinical Investigation*.

[45] Blanco-Melo D., Nilsson-Payant B. E., Liu W. C. (2020). Imbalanced host response to SARS-CoV-2 drives development of COVID-19. *Cell*.

[46] Desvergne B., Michalik L., Wahli W. (2006). Transcriptional regulation of metabolism. *Physiological Reviews*.

[47] Lee T. I., Young R. A. (2013). Transcriptional regulation and its misregulation in disease. *Cell*.

[48] Cartwright T., Perkins N. D., L Wilson C. (2016). NFKB1: a suppressor of inflammation, ageing and cancer. *FEBS Journal*.

[49] Kasembeli M. M., Bharadwaj U., Robinson P., Tweardy D. J. (2018). Contribution of STAT3 to inflammatory and fibrotic diseases and prospects for its targeting for treatment. *International Journal of Molecular Sciences*.

[50] Schonthaler H. B., Guinea-Viniegra J., Wagner E. F. (2011). Targeting inflammation by modulating the Jun/AP-1 pathway. *Annals of the Rheumatic Diseases*.

[51] Fullard N., Wilson C. L., Oakley F. (2012). Roles of c-Rel signalling in inflammation and disease. *The International Journal of Biochemistry and Cell Biology*.

